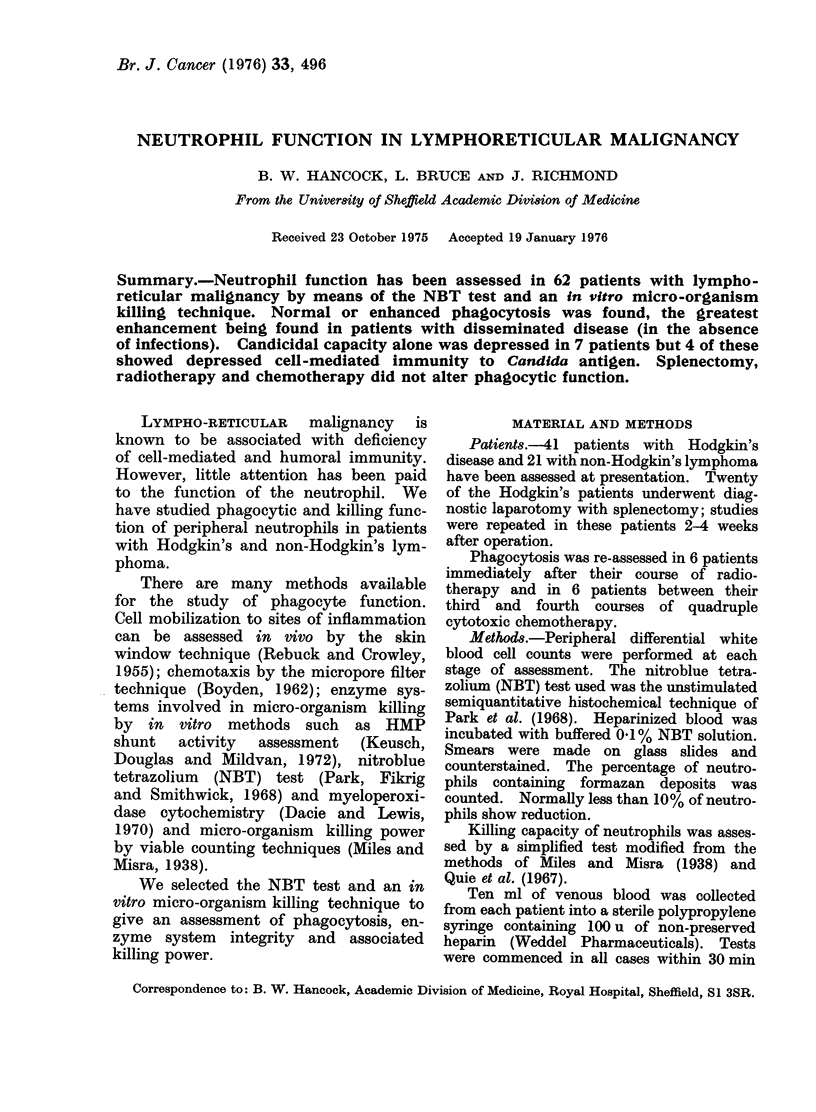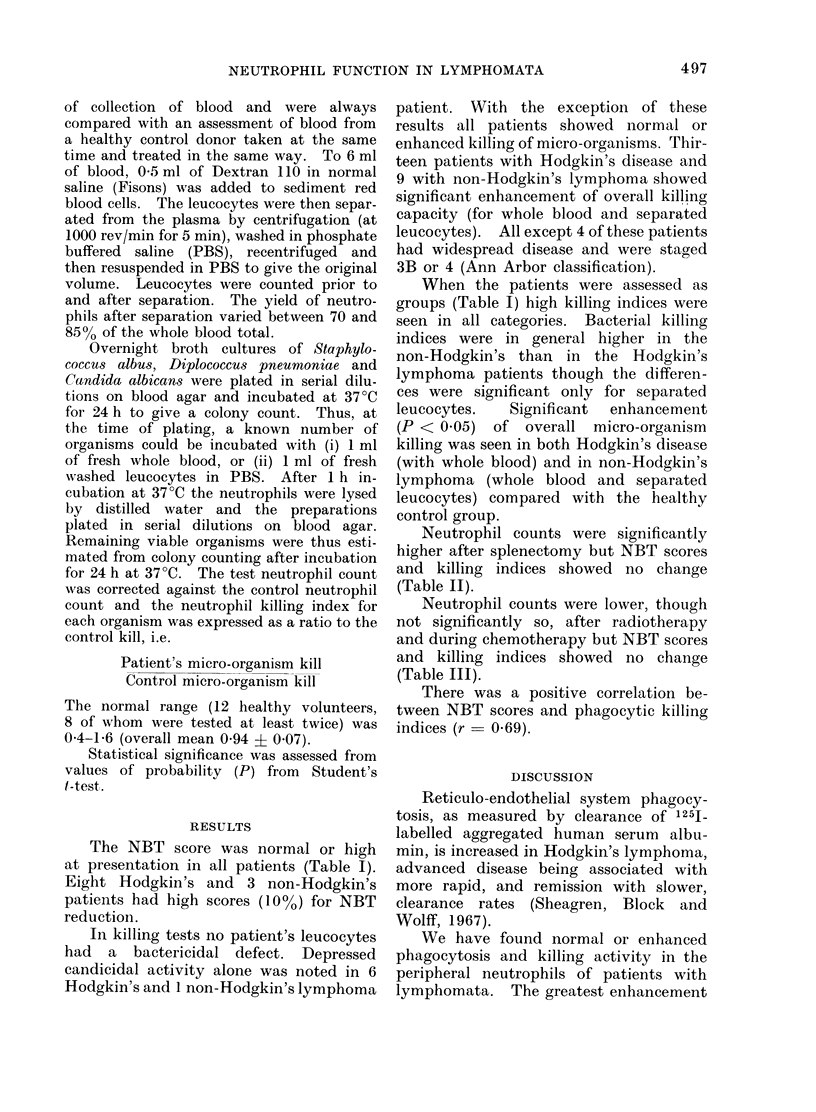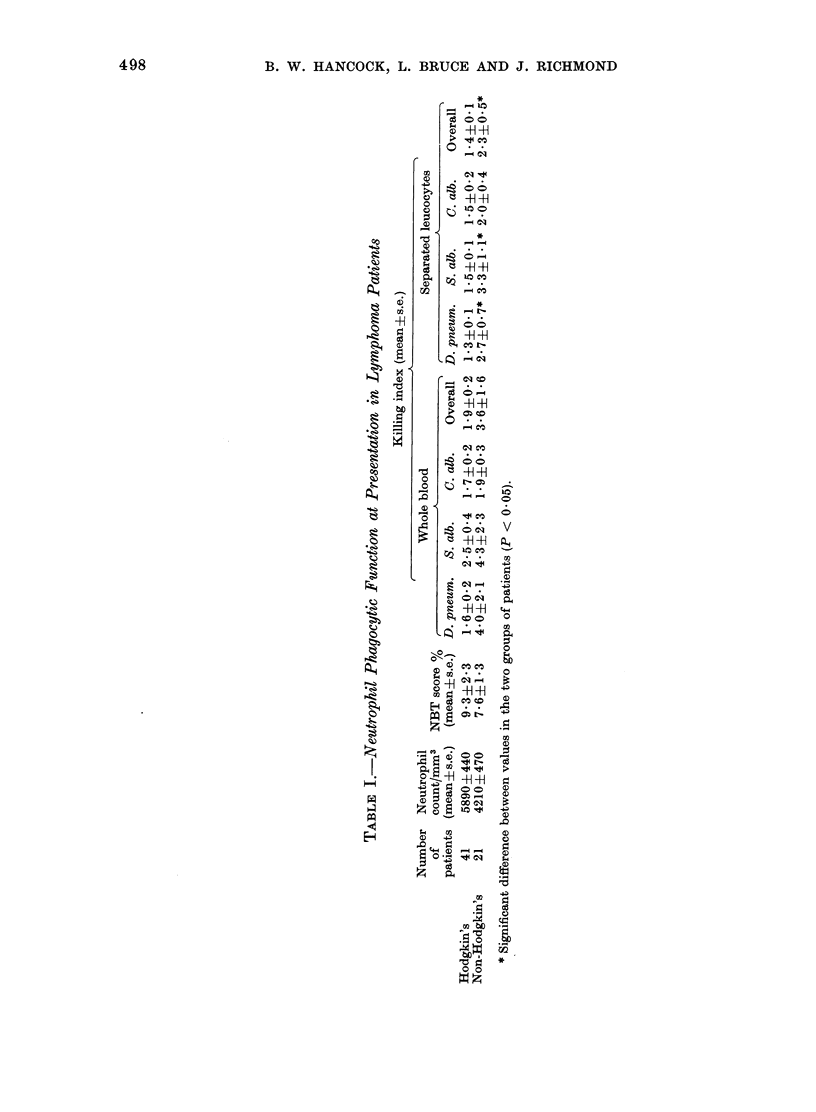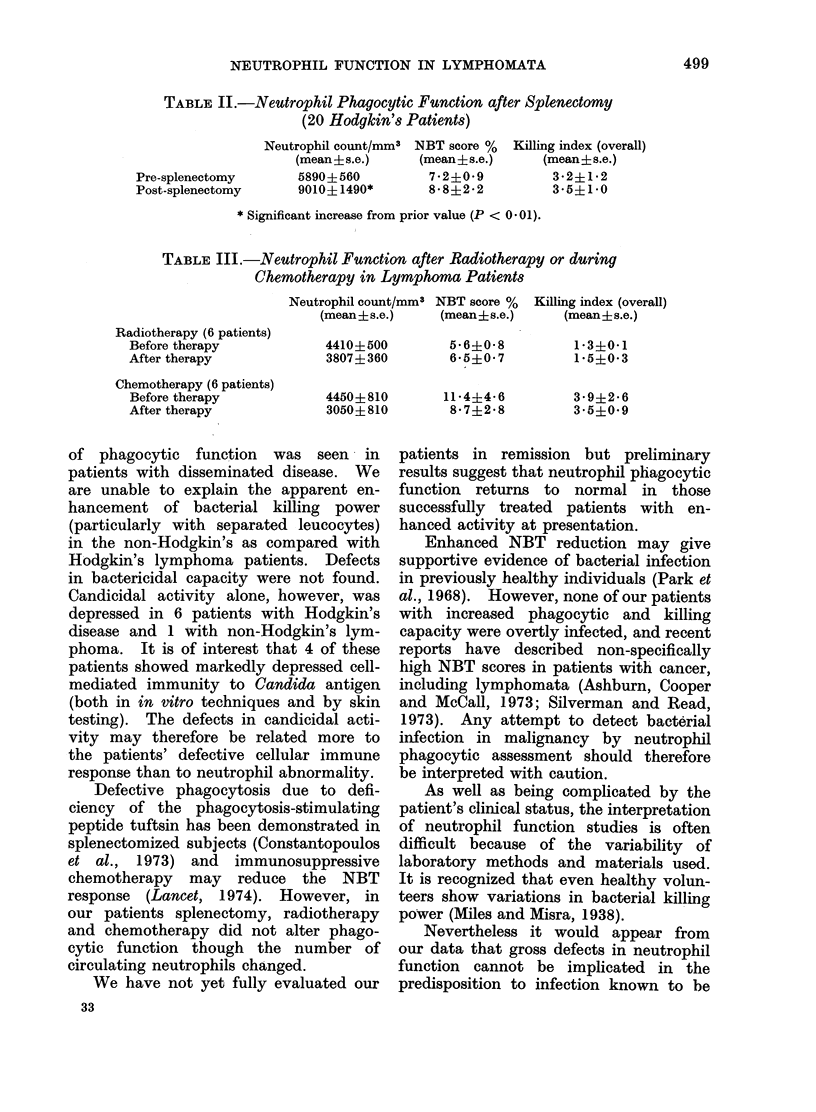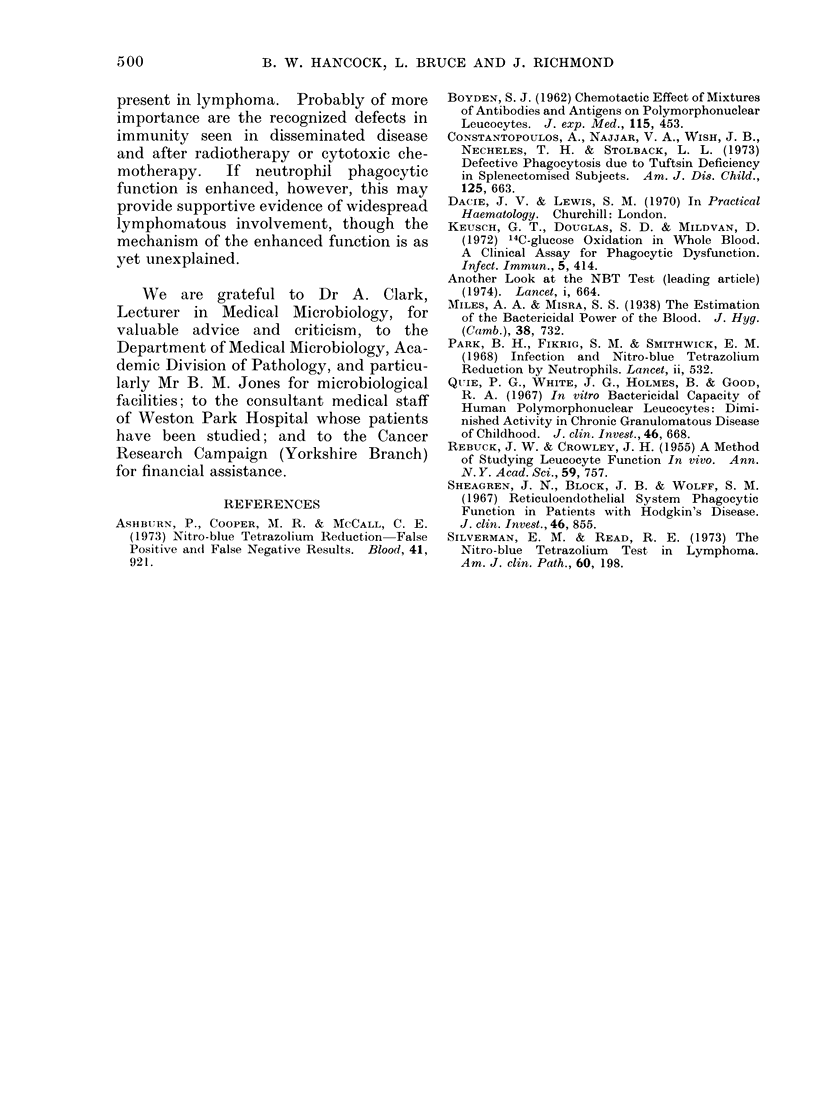# Neutrophil function in lymphoreticular malignancy.

**DOI:** 10.1038/bjc.1976.80

**Published:** 1976-05

**Authors:** B. W. Hancock, L. Bruce, J. Richmond

## Abstract

Neutrophil function has been assessed in 62 patients with lymphoreticular malignancy by means of the NBT test and an in vitro micro-organism killing technique. Normal or enhanced phagocytosis was found, the greatest enhancement being found in patients with disseminated disease (in the absence of infections). Candicidal capacity alone was depressed in 7 patients but 4 of these showed depressed cell-mediated immunity to Candida antigen. Splenectomy, radiotherapy and chemotherapy did not alter phagocytic function.


*Br. J. Cancer* (1976) **33**, 496

### NEUTROPHIL FUNCTION IN LYMPHORETICULAR MALIGNANCY

B. W. HANCOCK, L. BRUCE AND J. RICHMOND

*From the University of Sheffield Academic Division of Medicine*

Received 23 October 1975 Accepted 19 January 1976

**Summary.—Neutrophil function has been assessed in 62 patients with lymphoreticular malignancy by means of the NBT test and an *in vitro* micro-organism killing technique. Normal or enhanced phagocytosis was found, the greatest enhancement being found in patients with disseminated disease (in the absence of infections). Candicidal capacity alone was depressed in 7 patients but 4 of these showed depressed cell-mediated immunity to *Candida* antigen. Splenectomy, radiotherapy and chemotherapy did not alter phagocytic function.**

LYMPHO-RETICULAR malignancy is known to be associated with deficiency of cell-mediated and humoral immunity. However, little attention has been paid to the function of the neutrophil. We have studied phagocytic and killing function of peripheral neutrophils in patients with Hodgkin's and non-Hodgkin's lymphoma.

There are many methods available for the study of phagocyte function. Cell mobilization to sites of inflammation can be assessed *in vivo* by the skin window technique (Rebuck and Crowley, 1955); chemotaxis by the micropore filter technique (Boyden, 1962); enzyme systems involved in micro-organism killing by *in vitro* methods such as HMP shunt activity assessment (Keusch, Douglas and Mildvan, 1972), nitroblue tetrazolium (NBT) test (Park, Fikrig and Smithwick, 1968) and myeloperoxidase cytochemistry (Dacie and Lewis, 1970) and micro-organism killing power by viable counting techniques (Miles and Misra, 1938).

We selected the NBT test and an *in vitro* micro-organism killing technique to give an assessment of phagocytosis, enzyme system integrity and associated killing power.

### MATERIAL AND METHODS

*Patients.*—41 patients with Hodgkin's disease and 21 with non-Hodgkin's lymphoma have been assessed at presentation. Twenty of the Hodgkin's patients underwent diagnostic laparotomy with splenectomy; studies were repeated in these patients 2–4 weeks after operation.

Phagocytosis was re-assessed in 6 patients immediately after their course of radiotherapy and in 6 patients between their third and fourth courses of quadruple cytotoxic chemotherapy.

*Methods.*—Peripheral differential white blood cell counts were performed at each stage of assessment. The nitroblue tetrazolium (NBT) test used was the unstimulated semiquantitative histochemical technique of Park *et al.* (1968). Heparinized blood was incubated with buffered 0·1% NBT solution. Smears were made on glass slides and counterstained. The percentage of neutrophils containing formazan deposits was counted. Normally less than 10% of neutrophils show reduction.

Killing capacity of neutrophils was assessed by a simplified test modified from the methods of Miles and Misra (1938) and Quie *et al.* (1967).

Ten ml of venous blood was collected from each patient into a sterile polypropylene syringe containing 100 u of non-preserved heparin (Weddel Pharmaceuticals). Tests were commenced in all cases within 30 min

Correspondence to: B. W. Hancock, Academic Division of Medicine, Royal Hospital, Sheffield, S1 3SR.

of collection of blood and were always compared with an assessment of blood from a healthy control donor taken at the same time and treated in the same way. To 6 ml of blood, 0·5 ml of Dextran 110 in normal saline (Fisons) was added to sediment red blood cells. The leucocytes were then separated from the plasma by centrifugation (at 1000 rev/min for 5 min), washed in phosphate buffered saline (PBS), recentrifuged and then resuspended in PBS to give the original volume. Leucocytes were counted prior to and after separation. The yield of neutrophils after separation varied between 70 and 85% of the whole blood total.

Overnight broth cultures of *Staphylococcus albus*, *Diplococcus pneumoniae* and *Candida albicans* were plated in serial dilutions on blood agar and incubated at 37°C for 24 h to give a colony count. Thus, at the time of plating, a known number of organisms could be incubated with (i) 1 ml of fresh whole blood, or (ii) 1 ml of fresh washed leucocytes in PBS. After 1 h incubation at 37°C the neutrophils were lysed by distilled water and the preparations plated in serial dilutions on blood agar. Remaining viable organisms were thus estimated from colony counting after incubation for 24 h at 37°C. The test neutrophil count was corrected against the control neutrophil count and the neutrophil killing index for each organism was expressed as a ratio to the control kill, i.e.

$$\frac{\text{Patient's micro-organism kill}}{\text{Control micro-organism kill}}$$

The normal range (12 healthy volunteers, 8 of whom were tested at least twice) was 0·4–1·6 (overall mean 0·94 ± 0·07).

Statistical significance was assessed from values of probability ($P$) from Student's $t$-test.

### RESULTS

The NBT score was normal or high at presentation in all patients (Table I). Eight Hodgkin's and 3 non-Hodgkin's patients had high scores (10%) for NBT reduction.

In killing tests no patient's leucocytes had a bactericidal defect. Depressed candicidal activity alone was noted in 6 Hodgkin's and 1 non-Hodgkin's lymphoma patient. With the exception of these results all patients showed normal or enhanced killing of micro-organisms. Thirteen patients with Hodgkin's disease and 9 with non-Hodgkin's lymphoma showed significant enhancement of overall killing capacity (for whole blood and separated leucocytes). All except 4 of these patients had widespread disease and were staged 3B or 4 (Ann Arbor classification).

When the patients were assessed as groups (Table I) high killing indices were seen in all categories. Bacterial killing indices were in general higher in the non-Hodgkin's than in the Hodgkin's lymphoma patients though the differences were significant only for separated leucocytes. Significant enhancement ($P < 0{\cdot}05$) of overall micro-organism killing was seen in both Hodgkin's disease (with whole blood) and in non-Hodgkin's lymphoma (whole blood and separated leucocytes) compared with the healthy control group.

Neutrophil counts were significantly higher after splenectomy but NBT scores and killing indices showed no change (Table II).

Neutrophil counts were lower, though not significantly so, after radiotherapy and during chemotherapy but NBT scores and killing indices showed no change (Table III).

There was a positive correlation between NBT scores and phagocytic killing indices ($r = 0{\cdot}69$).

### DISCUSSION

Reticulo-endothelial system phagocytosis, as measured by clearance of $^{125}$I-labelled aggregated human serum albumin, is increased in Hodgkin's lymphoma, advanced disease being associated with more rapid, and remission with slower, clearance rates (Sheagren, Block and Wolff, 1967).

We have found normal or enhanced phagocytosis and killing activity in the peripheral neutrophils of patients with lymphomata. The greatest enhancement

TABLE I.—*Neutrophil Phagocytic Function at Presentation in Lymphoma Patients*

| | Number of patients | Neutrophil count/mm³ (mean±s.e.) | NBT score % (mean±s.e.) | Killing index (mean±s.e.) Whole blood | | | | Separated leucocytes | | | |
|---|---|---|---|---|---|---|---|---|---|---|---|
| | | | | *D. pneum.* | *S. alb.* | *C. alb.* | Overall | *D. pneum.* | *S. alb.* | *C. alb.* | Overall |
| Hodgkin's | 41 | 5890±440 | 9·3±2·3 | 1·6±0·2 | 2·5±0·4 | 1·7±0·2 | 1·9±0·2 | 1·3±0·1 | 1·5±0·1 | 1·5±0·2 | 1·4±0·1 |
| Non-Hodgkin's | 21 | 4210±470 | 7·6±1·3 | 4·0±2·1 | 4·3±2·3 | 1·9±0·3 | 3·6±1·6 | 2·7±0·7* | 3·3±1·1* | 2·0±0·4 | 2·3±0·5* |

* Significant difference between values in the two groups of patients ($P < 0·05$).

TABLE II.—*Neutrophil Phagocytic Function after Splenectomy (20 Hodgkin's Patients)*

| | Neutrophil count/mm³ (mean ± s.e.) | NBT score % (mean ± s.e.) | Killing index (overall) (mean ± s.e.) |
|---|---|---|---|
| Pre-splenectomy | 5890 ± 560 | 7·2 ± 0·9 | 3·2 ± 1·2 |
| Post-splenectomy | 9010 ± 1490* | 8·8 ± 2·2 | 3·5 ± 1·0 |

* Significant increase from prior value ($P < 0{\cdot}01$).

TABLE III.—*Neutrophil Function after Radiotherapy or during Chemotherapy in Lymphoma Patients*

| | Neutrophil count/mm³ (mean ± s.e.) | NBT score % (mean ± s.e.) | Killing index (overall) (mean ± s.e.) |
|---|---|---|---|
| Radiotherapy (6 patients) | | | |
| Before therapy | 4410 ± 500 | 5·6 ± 0·8 | 1·3 ± 0·1 |
| After therapy | 3807 ± 360 | 6·5 ± 0·7 | 1·5 ± 0·3 |
| Chemotherapy (6 patients) | | | |
| Before therapy | 4450 ± 810 | 11·4 ± 4·6 | 3·9 ± 2·6 |
| After therapy | 3050 ± 810 | 8·7 ± 2·8 | 3·5 ± 0·9 |

of phagocytic function was seen in patients with disseminated disease. We are unable to explain the apparent enhancement of bacterial killing power (particularly with separated leucocytes) in the non-Hodgkin's as compared with Hodgkin's lymphoma patients. Defects in bactericidal capacity were not found. Candicidal activity alone, however, was depressed in 6 patients with Hodgkin's disease and 1 with non-Hodgkin's lymphoma. It is of interest that 4 of these patients showed markedly depressed cell-mediated immunity to *Candida* antigen (both in *in vitro* techniques and by skin testing). The defects in candicidal activity may therefore be related more to the patients' defective cellular immune response than to neutrophil abnormality.

Defective phagocytosis due to deficiency of the phagocytosis-stimulating peptide tuftsin has been demonstrated in splenectomized subjects (Constantopoulos *et al.*, 1973) and immunosuppressive chemotherapy may reduce the NBT response (*Lancet*, 1974). However, in our patients splenectomy, radiotherapy and chemotherapy did not alter phagocytic function though the number of circulating neutrophils changed.

We have not yet fully evaluated our patients in remission but preliminary results suggest that neutrophil phagocytic function returns to normal in those successfully treated patients with enhanced activity at presentation.

Enhanced NBT reduction may give supportive evidence of bacterial infection in previously healthy individuals (Park *et al.*, 1968). However, none of our patients with increased phagocytic and killing capacity were overtly infected, and recent reports have described non-specifically high NBT scores in patients with cancer, including lymphomata (Ashburn, Cooper and McCall, 1973; Silverman and Read, 1973). Any attempt to detect bacterial infection in malignancy by neutrophil phagocytic assessment should therefore be interpreted with caution.

As well as being complicated by the patient's clinical status, the interpretation of neutrophil function studies is often difficult because of the variability of laboratory methods and materials used. It is recognized that even healthy volunteers show variations in bacterial killing power (Miles and Misra, 1938).

Nevertheless it would appear from our data that gross defects in neutrophil function cannot be implicated in the predisposition to infection known to be present in lymphoma. Probably of more importance are the recognized defects in immunity seen in disseminated disease and after radiotherapy or cytotoxic chemotherapy. If neutrophil phagocytic function is enhanced, however, this may provide supportive evidence of widespread lymphomatous involvement, though the mechanism of the enhanced function is as yet unexplained.

We are grateful to Dr A. Clark, Lecturer in Medical Microbiology, for valuable advice and criticism, to the Department of Medical Microbiology, Academic Division of Pathology, and particularly Mr B. M. Jones for microbiological facilities; to the consultant medical staff of Weston Park Hospital whose patients have been studied; and to the Cancer Research Campaign (Yorkshire Branch) for financial assistance.

### REFERENCES

ASHBURN, P., COOPER, M. R. & McCALL, C. E. (1973) Nitro-blue Tetrazolium Reduction—False Positive and False Negative Results. *Blood*, **41**, 921.

BOYDEN, S. J. (1962) Chemotactic Effect of Mixtures of Antibodies and Antigens on Polymorphonuclear Leucocytes. *J. exp. Med.*, **115**, 453.

CONSTANTOPOULOS, A., NAJJAR, V. A., WISH, J. B., NECHELES, T. H. & STOLBACK, L. L. (1973) Defective Phagocytosis due to Tuftsin Deficiency in Splenectomised Subjects. *Am. J. Dis. Child.*, **125**, 663.

DACIE, J. V. & LEWIS, S. M. (1970) In *Practical Haematology*. Churchill: London.

KEUSCH, G. T., DOUGLAS, S. D. & MILDVAN, D. (1972) $^{14}$C-glucose Oxidation in Whole Blood. A Clinical Assay for Phagocytic Dysfunction. *Infect. Immun.*, **5**, 414.

Another Look at the NBT Test (leading article) (1974). *Lancet*, i, 664.

MILES, A. A. & MISRA, S. S. (1938) The Estimation of the Bactericidal Power of the Blood. *J. Hyg. (Camb.)*, **38**, 732.

PARK, B. H., FIKRIG, S. M. & SMITHWICK, E. M. (1968) Infection and Nitro-blue Tetrazolium Reduction by Neutrophils. *Lancet*, ii, 532.

QUIE, P. G., WHITE, J. G., HOLMES, B. & GOOD, R. A. (1967) *In vitro* Bactericidal Capacity of Human Polymorphonuclear Leucocytes: Diminished Activity in Chronic Granulomatous Disease of Childhood. *J. clin. Invest.*, **46**, 668.

REBUCK, J. W. & CROWLEY, J. H. (1955) A Method of Studying Leucocyte Function *In vivo*. *Ann. N.Y. Acad. Sci.*, **59**, 757.

SHEAGREN, J. N., BLOCK, J. B. & WOLFF, S. M. (1967) Reticuloendothelial System Phagocytic Function in Patients with Hodgkin's Disease. *J. clin. Invest.*, **46**, 855.

SILVERMAN, E. M. & READ, R. E. (1973) The Nitro-blue Tetrazolium Test in Lymphoma. *Am. J. clin. Path.*, **60**, 198.